# Sensitivity Profile for Orientation Selectivity in the Visual Cortex of Goggle-Reared Mice

**DOI:** 10.1371/journal.pone.0040630

**Published:** 2012-07-06

**Authors:** Takamasa Yoshida, Katsuya Ozawa, Shigeru Tanaka

**Affiliations:** Laboratory for Visual Neurocomputing, RIKEN Brain Science Institute, Wako, Saitama, Japan; Instituto de Neurociencias de Alicante UMH-CSIC, Spain

## Abstract

It has been widely accepted that ocular dominance in the responses of visual cortical neurons can change depending on visual experience in a postnatal period. However, experience-dependent plasticity for orientation selectivity, which is another important response property of visual cortical neurons, is not yet fully understood. To address this issue, using intrinsic signal imaging and two-photon calcium imaging we attempted to observe the alteration of orientation selectivity in the visual cortex of juvenile and adult mice reared with head-mounted goggles, through which animals can experience only the vertical orientation. After one week of goggle rearing, the density of neurons optimally responding to the exposed orientation increased, while that responding to unexposed orientations decreased. These changes can be interpreted as a reallocation of preferred orientations among visually responsive neurons. Our obtained sensitivity profile for orientation selectivity showed a marked peak at 5 weeks and sustained elevation at 12 weeks and later. These features indicate the existence of a critical period between 4 and 7 weeks and residual orientation plasticity in adult mice. The presence of a dip in the sensitivity profile at 10 weeks suggests that different mechanisms are involved in orientation plasticity in childhood and adulthood.

## Introduction

The brain circuits in the sensory cortices are shaped innately and later refined depending on animals' experience to achieve the efficient information processing of sensory stimuli. In particular, the most sensitive period for the plasticity of neuronal response specificity during early development is called the critical period. The plasticity of ocular dominance [1]–[7] and orientation selectivity [8]–[14] in the visual cortex has been studied intensively. Recently, the mechanisms of ocular dominance plasticity have been examined in detail using genetically manipulated mice as well as the wild-type reared under monocular deprivation [15]–[18]. Orientation plasticity, however, has not been studied as fully as ocular dominance [19], [20] because of the difficulty of visual manipulation by which animals are exposed to a specific orientation under freely moving conditions.

In our previous studies, to investigate the cortical plasticity of orientation selectivity in rodents, we fabricated miniature head-mounted goggles to impose single-orientation exposure on rats [21] and mice [22] based on the design of goggles for cats [23]. Our intrinsic signal imaging in the visual cortex of mice reared with the goggles has revealed that cortical orientation representation can change to exhibit a bias towards the exposed orientation [22], although the change was less conspicuous than that in goggle-reared kittens, which showed a marked overrepresentation of the exposed orientation. One of the remaining questions is whether the number of neurons optimally responding to the exposed orientation increases [12], the number of neurons optimally responding to the unexposed orientations decreases [11] or both [13], [14], [24]. One solution has been provided by cellular-level recordings obtained from two-photon calcium imaging [25]. Another question is whether or not the plasticity of orientation selectivity is preserved to some extent even in adult rodents, as observed for ocular dominance plasticity [26]–[32]. However, except for the reports of Frenkel et al. [19] and Treviño et al. [33], little evidence supporting adult orientation plasticity has been reported. To address this issue, we need to obtain an age-dependent sensitivity profile for orientation selectivity in mice. However, such a profile has only been obtained for cats [14].

In this study, to answer to the above-mentioned questions, we used intrinsic signal imaging and two-photon calcium imaging with goggle-reared mice. The intrinsic signal imaging showed that responses to the exposed orientation were relatively potentiated, whereas responses to unexposed orientations were depressed. The two-photon imaging revealed no difference in the number of visually responsive neurons between normally reared (NR) and goggle-reared (GR) mice, despite an increase in the number of neurons preferring the exposed orientation and a decrease in the number of neurons preferring unexposed orientations in GR mice. A horizontal bias in the orientation selectivity in NR mice irrespective of age was observed by the two imaging methods. The strong resemblance between orientation histograms for the relative size of activation areas measured by intrinsic signal imaging and for the relative number of active neurons measured by two-photon imaging indicates that intrinsic signals reflect population activities in the visual cortex. On the basis of this observation, we attempted to perform intrinsic signal imaging in a number of mice of different ages between postnatal 3 and 15 weeks to obtain a sensitivity profile for orientation selectivity, which was induced by goggle rearing for one week. The sensitivity was sufficiently high in the interval between 4 and 7 weeks to suggest that this is the critical period for orientation plasticity in the mouse visual cortex. Furthermore, we observed a significant increase in orientation plasticity even in the adult group, suggesting the presence of adult plasticity for orientation selectivity.

## Results

### Modification of orientation preference by vertical-orientation exposure

**Figure 1:**
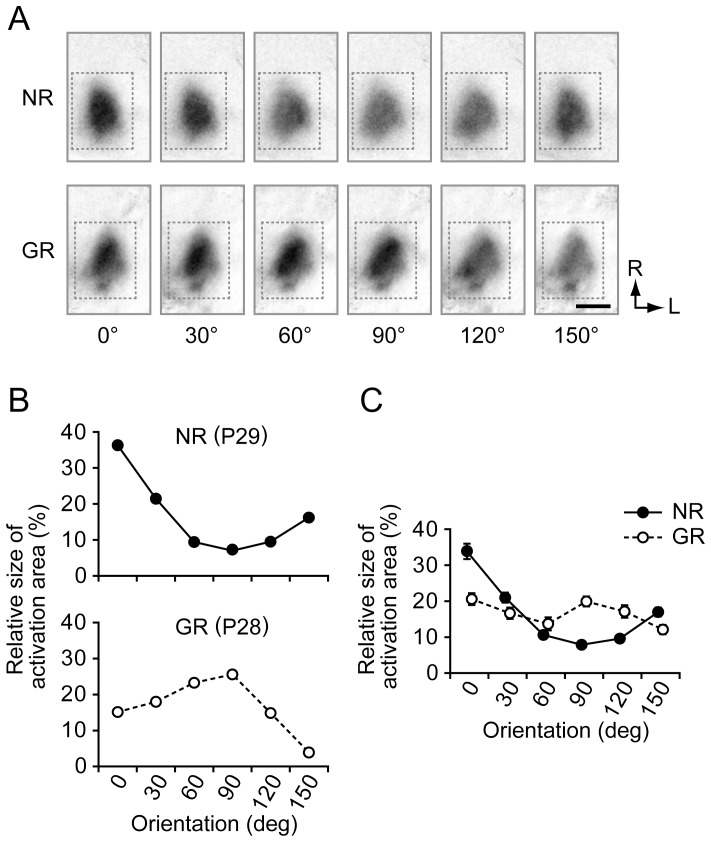
Cortical activation patterns and orientation distributions. (A) Single-orientation maps of an NR mouse and a mouse GR from P21 to P28, which were reconstructed from intrinsic signal optical imaging conducted at P29 and P28, respectively. The darkness indicates the strength of intrinsic signals evoked by oriented grating stimuli. The dotted rectangles show the region of interest. R: rostral, L: lateral. (B) Orientation distributions obtained from the single-orientation maps shown in (A). The ordinate indicates the relative size of activation areas eliciting stimulus-related responses stronger than 2SD for each stimulus orientation. (C) Group-averaged orientation distributions for NR (n = 45) and GR (n = 44) groups of juvenile mice. Error bars indicate SE (standard error). All scale bars indicate 1 mm.

Single-orientation maps obtained from typical NR and GR juvenile mice are shown in the top and bottom rows of Fig. 1A, respectively, where the darkness of the pixels indicates the strength of the stimulus-related response. The darkness of the activation area was different for different stimulus orientations in both NR and GR mice, which reflects the presence of an orientation representation bias in the region of interest (see Materials and Methods). In the NR mouse, the response strength to a stimulus orientation was strongest around the horizontal orientation. In contrast, in the GR mouse, the response strength was strongest around the vertical orientation. These trends can also be seen in the orientation distributions (Fig. 1B). The distribution for the NR mouse exhibited a maximum at 0° and a minimum at 90° (Fig. 1B top). For the GR mouse, the distribution had a peak at 90° (Fig. 1B bottom). The group-averaged orientation distributions of the relative size of activation areas in juvenile mice had a prominent peak at 0° for the NR group and a broad maximum at 90° for the GR group (Fig. 1C). The sum of the relative sizes of the activation areas for 60°, 90° and 120° in the juvenile GR group was significantly larger than that for the juvenile NR group (n = 45 (NR), n = 44 (GR), p = 2.44×10^−12^, Student t-test).

### Cellular-level modification of orientation representation

**Figure 2:**
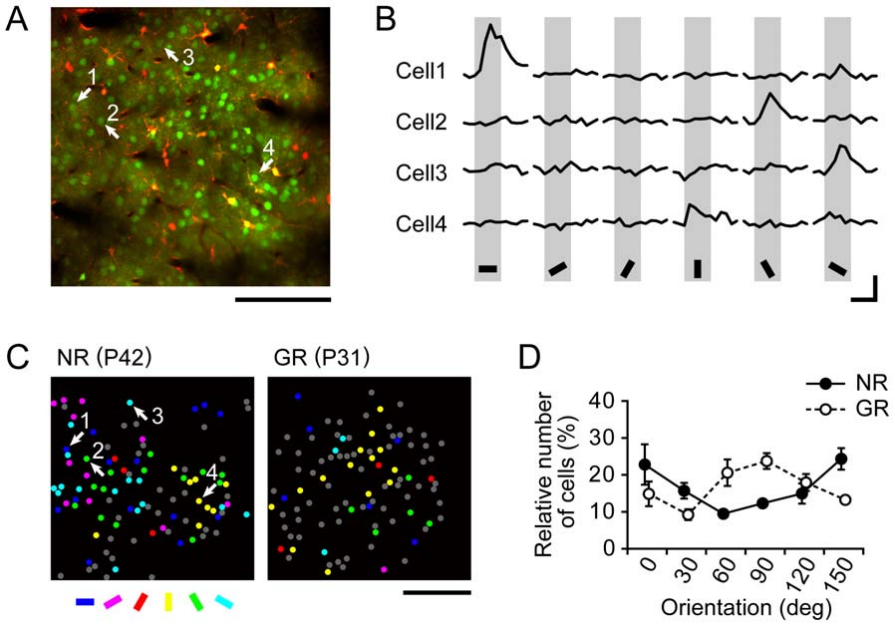
Typical results of two-photon imaging for juvenile NR and GR mice. (A) Image of Ca^2+^ signals in a slice of the VI of an NR mouse. Fluorescence signals from OGB-1 (green) and SR101 (orange) identify neurons and astrocytes, respectively. Dark spots and strips indicate blood vessels. The scale bar indicates 100 µm. (B) Traces of Ca^2+^ responses of four neurons to stimulus orientations of grating stimuli, where stimulus orientations are indicated by the inclinations of the black bars placed below. These four neurons were located at positions indicated by the numbered arrows in (A). Gray columns represent 5 s stimulation periods. Vertical and horizontal bars respectively indicate 5% (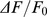) and 5 s. (C) Color-coded images of vigorously responsive neurons in single slices of an NR mouse (left) and a mouse GR from P23 to P31 (right), which were reconstructed from two-photon Ca^2+^ imaging conducted at P42 and P31, respectively. The left image was reconstructed from the Ca^2+^ signals in the slice shown in (A). The color of the dots indicates the preferred orientation, whereas gray dots indicate responsive but unoriented neurons. The color code is shown below the left image. (D) Group-averaged orientation distributions for NR (n = 3) and GR (n = 3) mice. Error bars indicate SE. The scale bar indicates 100 µm.

Images of several slices per animal (on average, three slices) at different depths from the cortical surface were recorded using *in vivo* two-photon imaging. An image of Ca^2+^ signals recorded at a depth of 230 µm in an NR mouse is shown as a typical example in Fig. 2A. Neurons and astrocytes exhibited different colors corresponding to the different wavelengths of emission light from OGB-1 (green) and SR101 (red), respectively (see Materials and Methods). Examples of the time courses of Ca^2+^ signals at four cells in response to each stimulus orientation are shown in Fig. 2B. Cells 1, 2, 3 and 4 were preferential (OSI = 0.52, 0.34, 0.23 and 0.21, see Materials and Methods) for orientations of 0°, 120°, 150° and 90°, respectively. Figure 2C shows only visually responsive neurons for typical NR and GR mice in single planes recorded at depths of 230 µm and 150 µm in the left and right panels, respectively. The color of the dots represents the preferred orientation of neurons, and the gray dots represent unoriented neurons. The number of light blue dots was largest (15 of 66 oriented neurons; 22.7%) in the plane of the NR mouse. In contrast, the number of yellow dots was largest (14 of 32 oriented neurons; 43.8%) in the plane of the GR mouse. It can be observed that cellular-level orientation representations in the NR and GR mice, respectively, tended to be biased towards the horizontal and vertical orientations. These tendencies were supported by the group-averaged orientation distributions in NR and GR mice (Fig. 2D). Note that Fig. 2D has a reasonably strong resemblance to Fig. 1C.

**Figure 3:**
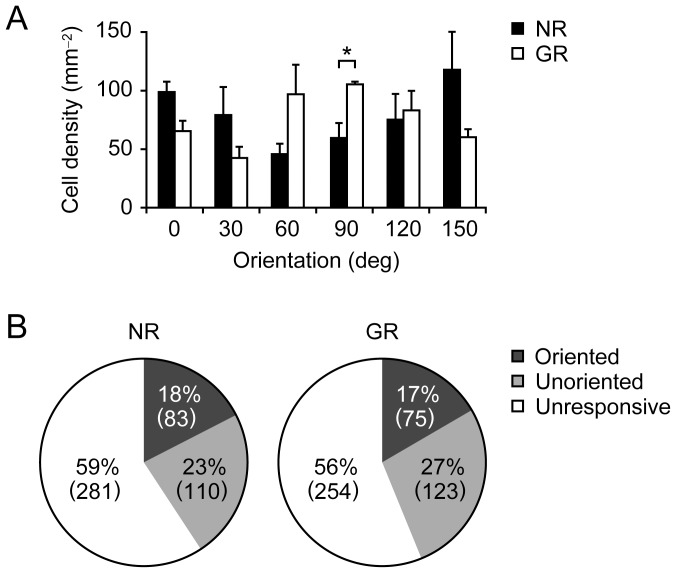
Neuron densities and abundance ratios of oriented and unoriented neurons. (A) Histograms of neuron density for NR and GR mice. The difference in the neuron density between NR and GR mice was statistically significant for the vertical orientation (p<0.05, Student t-test). Error bars indicate SE. (B) Ratios of the numbers of oriented, unoriented and unresponsive neurons to the total number of neurons identified by OGB-1 signals for the NR group (left) and GR group (right). The absolute numbers of respective types of neurons are shown in parentheses. The abundance ratio of oriented neurons as well as that of unoriented neurons did not differ between the two groups.

Together with the findings obtained from intrinsic signal imaging, it was demonstrated that the cortical representation of the exposed vertical orientation became predominant over the representation of unexposed orientations after one week of continuous vertical-orientation exposure not only at the population level but also at the cellular level. This raises the question of whether such overrepresentation of the exposed orientation is attributable to an increase in the number of neurons preferring the exposed orientation or a decrease in the number of neurons preferring unexposed orientations with the number of neurons preferring the exposed orientation remaining unchanged. To answer this question, we estimated the densities of neurons selective for each orientation for the NR and GR groups of juvenile mice, as shown in Fig. 3A. Comparison of the cell density histograms between the NR and GR groups demonstrated that the average densities at 60°, 90° and 120° in the GR group were greater than those in the NR group, and statistical significance was confirmed for 90° (p = 0.0260, Student t-test). Also, the average densities at 0°, 30° and 150° in the NR group were greater than those in the GR group, although no statistical significance was observed. In this analysis, the total numbers of oriented cells in the NR and GR groups were 474 and 452, respectively, which are comparable numbers. Moreover, the ratios of the number of oriented neurons to that of all stained neurons in the NR and GR groups were 17.5±3.3(SE, standard error) % and 16.6±2.0(SE) %, respectively, with no significant difference (Fig. 3B). The proportions of visually responsive neurons in the NR and GR groups were 40.8±10.8(SE) % and 43.8±5.8(SE) %, respectively, which also showed no significant difference. That is, the number of visually responsive neurons as well as the number of oriented neurons did not differ significantly between the two groups. These findings from cell density analysis lead to the conclusion that the one week of continuous goggle rearing of juvenile mice increased the number of neurons selective for the exposed vertical orientation without changing the number of visually responsive neurons or the number of oriented neurons.

### Age dependence of changes in the orientation bias

Having confirmed that the orientation distributions of neuronal populations obtained from intrinsic signal imaging were strongly correlated with those of single neurons measured by two-photon imaging, we investigated the changes in the orientation representation bias induced by the one week of goggle rearing of mice at different ages. To evaluate the bias of the orientation representation towards the horizontal or vertical orientation, we defined the orientation bias index (OBI) as
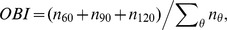
where 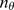 is the number of pixels strongly responding to 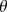 in intrinsic signal imaging or the number of neurons preferring orientation 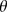 in two-photon imaging. When the OBI is smaller or larger than 0.5, the orientation is biased towards the horizontal or vertical orientation, respectively. We also defined the sensitivity index (SI) as a function of age as
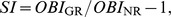
where 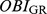 and 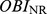 are the OBIs for GR and NR mice at the same age, respectively. Therefore, a positive SI indicates that vertical-orientation exposure enhances the orientation representation bias toward the vertical orientation.

**Figure 4:**
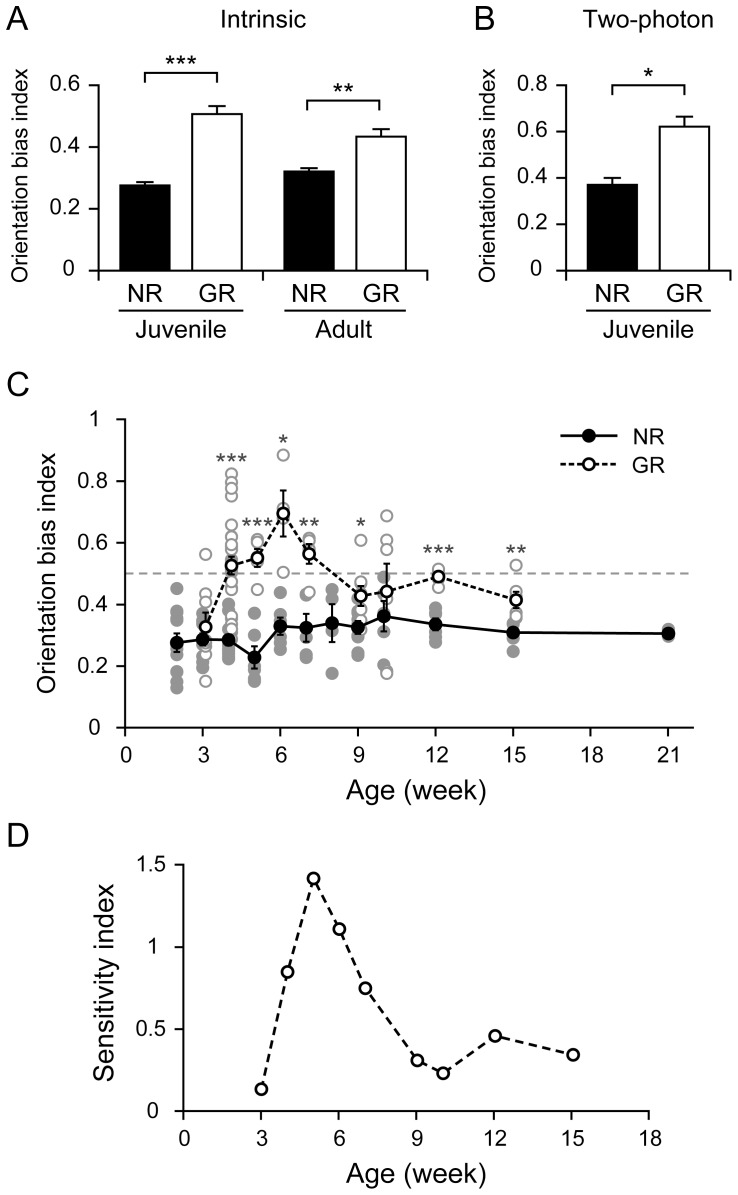
Orientation bias indices (OBIs) for NR and GR mice and sensitivity profile for orientation plasticity. (A) Averaged OBIs obtained from intrinsic signal imaging of NR and GR groups for juvenile and adult mice. (B) Averaged OBIs obtained from two-photon imaging of juvenile NR and GR groups. (C) Age dependence of OBIs averaged among animals of the same age for NR and GR mice. The dashed gray line indicates OBI = 0.5, corresponding to the absence of any representation bias towards the vertical or horizontal orientation. Faint dots indicate OBIs for individual mice. Error bars indicate SE. Significant differences in OBIs for GR and NR mice are shown as *p<0.05, **p<0.01 and ***p<0.001. (D) Sensitivity profile for orientation plasticity induced by the one-week of exposure to vertical orientation from 3w to 15w. The critical period for orientation plasticity is suggested to lie between 4w and 7w, during which the sensitivity index (SI) is prominently large. Also, orientation plasticity is found to be preserved to some extent even after 12w.

Significant differences in the OBIs estimated from the intrinsic signals were found between the NR and GR groups in both juvenile and adult mice (n = 45 (juvenile NR), n = 44 (juvenile GR), n = 35 (adult NR), n = 26 (adult GR), p = 4.69×10^−13^ for juvenile NR/juvenile GR; p = 1.31×10^−3^ for adult NR/adult GR; p = 0.278 for juvenile NR/adult NR; p = 0.108 for juvenile GR/adult GR; Tukey HSD test, Fig. 4A). For juvenile mice, the OBIs estimated from Ca^2+^ signals also exhibited a significant difference between the NR and GR groups (n = 3 (juvenile NR), n = 3 (juvenile GR), p = 0.0115, Student t-test, Fig. 4B). The values of OBI and SI are plotted from 3w to 15w in Figs. 4C and 4D, respectively. The age-dependent profile of the mean OBI for GR mice had a peak at 6w, whereas that for NR mice was flat (F_11,71_ = 1.32, p = 0.233, ANOVA, Fig. 4C, filled circles). The differences in the mean OBIs for the NR and GR mice were significant except at 3w and 10w (n = 10 (NR), n = 8 (GR), p = 0.399 for 3w; n = 11 (NR), n = 27 (GR), p = 1.00×10^−5^ for 4w; n = 6 (NR), n = 5 (GR), p = 6.84×10^−5^ for 5w; n = 6 (NR), n = 4 (GR), p = 0.0128 for 6w; n = 5 (NR), n = 5 (GR), p = 3.26×10^−3^ for 7w; n = 9 (NR), n = 8 (GR), p = 0.0207 for 9w; n = 5 (NR), n = 6 (GR), p = 0.458 for 10w; n = 6 (NR), n = 6 (GR), p = 2.43×10^−5^ for 12w; n = 6 (NR), n = 6 (GR), p = 9.00×10^−3^ for 15w, Student t-test, Fig. 4C) with marked differences in the interval between 4w and 7w. The sensitivity profile for orientation selectivity had a prominent peak at 5w (Fig. 4D). This profile suggests that there is a critical period for orientation plasticity between 4w and 7w. Interestingly, the SI increased again after 12w, suggesting that orientation plasticity is preserved to some extent even in adulthood. These findings indicate that continuous vertical-orientation exposure for one week is able to significantly weaken the inherent horizontal bias in the orientation representation in the mouse V1.

## Discussion

In this study, we investigated the alteration of orientation selectivity in the mouse visual cortex under continuous orientation-restricted visual experience with head-mounted goggles. As a result, we found that goggle rearing changed the innate horizontal orientation bias to the overrepresentation of the exposed orientation. This change was observed in the proportion of oriented neurons in GR mice, whereas the total number of oriented neurons in NR mice was preserved. We further obtained a sensitivity profile for orientation selectivity in GR mice of different ages, which revealed the presence of a critical period for orientation selectivity and subsequent adult plasticity.

### Innate horizontal bias in NR mice

A representation bias towards the horizontal orientation has been reported in electrophysiological studies on mice [34] and rats [35]. The distribution of the relative areas optimally responding to individual stimulus orientations in NR juvenile mice, which was observed by intrinsic signal imaging, exhibited a notable peak at the horizontal orientation (Fig. 1C), consistent with the observation by Cang et al. [36]. Also, the distribution of the relative number of neurons selective for stimulus orientations had a marked peak at the horizontal orientation in the two-photon imaging of juvenile mice (Fig. 2D), as reported by Mank et al. [37], Rochefort et al. [38] and Kreile et al. [25]. These observations strongly support the existence of horizontal bias in the visual cortex of juvenile mice. Furthermore, we found that the horizontal bias did not change with postnatal age (Fig. 4C). This was supported by the insignificant differences in the OBIs between juvenile and adult mice (Fig. 4A). The horizontal bias in the adult mouse visual cortex is a distinct feature different from the horizontal bias in the cat visual cortex, which was observed only immediately after the orientation map emerged but then disappeared [14]. Frenkel et al. [19] also reported the presence of a horizontal bias in adult mice using visually evoked potential recording, although they did not find a horizontal bias in juvenile mice. Their failure to observe a horizontal bias in juvenile mice may be due to the sampling bias inherent in their recording method.

Kreile et al. [25] reported that the horizontal bias depended on the cortical depth in layer 2/3: the bias is stronger in upper parts and weaker in lower parts. We conducted two-photon imaging at different cortical depths but we did not sample neurons systematically, as Kreile et al. did. The average depths of our imaging of NR and GR mice were 176.5±13.8 (SE) µm and 141.3±8.4 (SE) µm, respectively. According to the sub-layer categories of recording depths in Kreile et al., it is quite likely that our imaging was conducted at the upper part of layer 2/3 for both NR and GR mice. Our observation of the strong orientation bias in NR mice was consistent with their observation at the upper part of layer 2/3. However, in our imaging, we rarely observed the orientation bias toward not only horizontal but also vertical orientations in their NR mice. Our orientation bias was almost exclusively toward horizontal orientation, as consistent with the bias observed in our optical imaging of intrinsic signals reflecting population activities in a large area of the primary visual cortex. It is likely that the small window size in two-photon imaging gives rise to a sampling bias that may produce a feigned orientation bias. In this sense, it may be that the results of Kreile et al. with a window of 180×180 µm are more susceptible to a sampling bias than ours with a window of 300×300 µm.

### Representation bias towards the exposed orientation in GR mice

The results of intrinsic signal imaging and two-photon imaging showed a change in the orientation bias towards the exposed vertical orientation in the mouse visual cortex in an experience-dependent manner upon continuous single-orientation exposure for one week. The same phenomenon has been reported in mice [22], [25], rats [21] and cats [12]–[14], [24]. In particular, Kreile et al. [25] used orientation-restricting goggles similar to ours to expose mice to a single orientation and examined the impact of the altered visual experience on orientation selectivity modification using two-photon imaging. As a result, they demonstrated that the relative number of responsive neurons at the exposed orientation was larger in GR mice than in NR mice and that the relative number of at unexposed orientations was smaller in GR mice than in NR mice. This finding is reasonably consistent with the present results. However, they found that such changes in neuronal responses to single-orientation exposure were limited to the lower part of layer 2/3 (300–340 µm), whereas we observed similar changes in the upper part of layer 2/3. The origin of this discrepancy is unclear.

We also analyzed the density of neurons for individual stimulus orientations. The neuron density around the exposed orientation was higher in GR mice than in NR mice, whereas the density at the other orientations tended to be lower in GR mice than in NR mice. In addition, there were no differences in the absolute number of oriented neurons or unoriented visually responsive neurons between NR and GR mice. Kreile et al. [25] also reported that the abundance ratio of visually responsive neurons to all neurons identified by two-photon imaging was not significantly different between the NR and GR mice, consistent with our observation. However, they found that about 70% of the neurons in the mouse visual cortex were responsive, which greatly deviated from our measured result. This disagreement may be attributed to differences in the spatial and temporal frequencies of the stimulus gratings, because the abundance ratio of responsive neurons in the present study (NR mice, 41%; GR mice, 44%) was close to those reported in previous studies using the same stimulus parameters: 37% (rats) in Ohki et al. [39] and 35% (mice) in Sohya et al. [40].

The above discussion suggests that continuous single-orientation exposure with head-mounted goggles induces a reallocation of the orientation preference among visually responsive neurons. These observations indicate that the increase in the number of neurons selective for the exposed orientation is balanced with the decrease in the number of neurons selective for unexposed orientations. There has been debate as to whether the number of neurons preferring the exposed orientation increases (instruction mechanism) [8], [41] or the number of neurons originally selective for unexposed orientations decreases while the number of neurons preferring the exposed orientation remains unchanged (selection mechanism) in the cat visual cortex [11], [42]. The present study together with Kreile et al.'s study settles this debate. Namely, the instruction mechanism occurs in practice, although the selection mechanism cannot be ruled out.

Moreover, Kreile et al. [25] reported that in the upper part of layer 2/3, not only the number of neurons optimally responding to unexposed orientations but also that responding to the exposed orientation decreased. However, no such effect was observed in our two-photon imaging conducted in the upper parts of layer 2/3. This discrepancy may result from the different durations of goggle rearing: Kreile et al. conducted goggle rearing for three weeks, compared with one week in this study. When intrinsic signal imaging was performed in GR kittens, longer durations of goggle rearing tended to reduce neural responses to all oriented visual stimuli as well as the degree of overrepresentation of the exposed orientation [13]. It is speculated that regardless of the species, the neuronal responses in the upper parts of layer 2/3 tend to decrease owing to longer-term single-orientation exposure, which is equivalent to the longer-term deprivation of stimulus orientations except a single orientation.

### Methodological differences in the orientation exposure

Wang et al. [20] reported that adult mice placed in a striped environment for 15 min/day and in darkness at other times had enhanced response reliability at the exposed orientation but a reduced number of orientation-selective neurons. In particular, the difference in the number of orientation-selective neurons between their results and our observations may be due to the different durations of single-orientation exposure and the intervention of dark rearing. It may also be related to the adaptation of neuronal activity in response to a briefly experienced orientation, as observed in adult cats [43]. In addition, the behavioral relevance of visual experience may be relevant to orientation plasticity: In the experiment performed by Wang et al., animals were passively exposed to a single oriented grating displayed on a monitor screen while being constrained in a bag. In contrast, our GR mice were able to actively explore a rich environment through the orientation-restricting goggles. Regarding this matter, Li et al.'s experiment [44] is suggestive: anesthetized and paralyzed ferrets passively exposed to a one-way moving oriented grating did not exhibit any changes in preferred orientations, although their preferred directions of motion changed towards the exposed direction.

### Species differences in the degree of overrepresentation of the exposed orientation

The peaks of the orientation distributions around the exposed orientation in the GR mice were much lower than those in cats reared with similar goggles for a comparable duration [13], [14]. From the theoretical viewpoint, short-range excitatory interaction among neurons produces the clustering of preferred orientations, and longer-range lateral inhibition delimits the cluster size, resulting in orderly arrangements of preferred orientations [45], [46]. The absence of regular arrangements of preferred orientations in the rodent visual cortex [39] implies that the short-range excitatory interaction is weak. In particular, the weak excitatory interaction may reduce the expansion of clusters representing similar orientations, resulting in the small increase in the number of pixels selective for the exposed orientation. Therefore, the peak of orientation distributions at the exposed orientation in mice may not be as high as that in cats which have orderly orientation maps.

### Sensitivity profile for orientation selectivity

Although the existence of a critical period for ocular dominance plasticity was first demonstrated in the mouse visual cortex by Gordon and Stryker [7], little is known about the critical period for orientation plasticity in mice. Age-dependent changes in orientation selectivity have been investigated in cats [47] and ferrets [48], [49], but the sensitivity profile for orientation selectivity has only been reported for the cat visual cortex [14]. In the present study, we were able to obtain a sensitivity profile for orientation selectivity in the mouse visual cortex for the first time, and we suggest that the critical period for orientation plasticity ranges between postnatal 4w and 7w. This period is later than the critical period for ocular dominance plasticity, which begins after eye opening, peaks around 4w and ends at P32 in the case of four-day monocular deprivation [7]. Although the duration of monocular deprivation in their study was different from that of single-orientation exposure in the present study, the retardation of the orientation critical period may be due to different cortical layers and/or different molecular mechanisms responsible for ocular dominance plasticity and orientation plasticity.

Furthermore, the sensitivity profile indicated that orientation plasticity remains to some degree even after the critical period. In rodent studies, adult plasticity of ocular dominance induced by monocular deprivation [26]–[32] and adult plasticity of retinotopy induced by retinal lesion or removing the contralateral eye [50], [51] have been investigated in depth. Although there have been a few studies on adult plasticity for orientation preference in the mouse visual cortex [19], [33], the present work is the first to provide solid evidence of adult orientation plasticity. Regarding ocular dominance, the mechanisms underlying the critical period plasticity and adult plasticity have been suggested to be different [16], [18], [32], [52]–[55]. The dip in the sensitivity profile around 10w may be related to the switch between different mechanisms of orientation plasticity, as suggested for ocular dominance plasticity.

## Materials and Methods

The surgery and recordings in the present study were approved by the Institutional Animal Research Committee of RIKEN and were performed in accordance with the guidelines of the Japanese Neuroscience Society.

### Animal preparations

We used C57BL/6J mice (n = 175) from postnatal day 15 (P15) to 36 weeks (36w). One hundred and sixty-nine mice (NR, n = 92; GR, n = 77) and six mice (NR, n = 3; GR, n = 3) were subjected to intrinsic signal optical imaging and two-photon calcium imaging, respectively. We defined animals younger than 7w as juvenile (n = 94) and animals older than 8w as adult (n = 61). All animals were raised in an environment in which toys, tunnels and running wheels were placed. Light (250–300 lx) and dark (70 lx) periods were alternated every 12 hours. The animals were divided into two groups: one group of animals were reared under normal visual conditions; the other group were reared with head-mounted goggles providing vertical-orientation exposure for one week immediately before optical imaging or two-photon imaging was performed, and at other times they were kept under normal visual conditions without goggles.

### Goggle mounting

**Figure 5:**
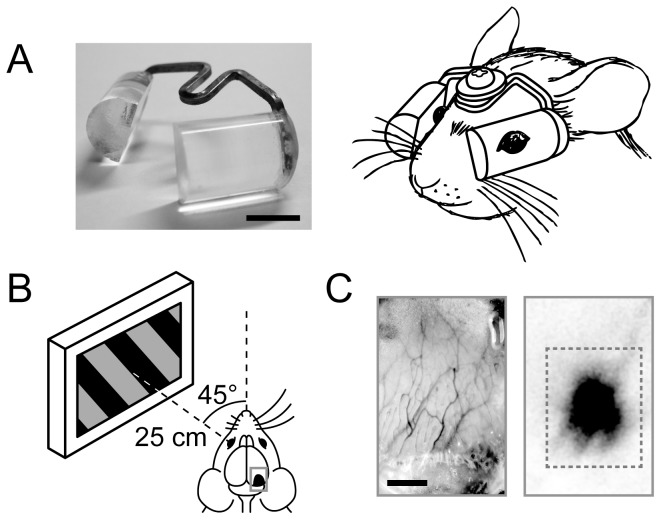
Orientation-restricting goggles and experimental setup for intrinsic signal optical imaging. (A) Photograph of goggles (left) and picture of a goggle-mounted mouse (right). The scale bar indicates 5 mm. (B) The monitor screen presenting visual stimuli was placed 25 cm apart from either eye of a mouse. The angle between the center of the screen and the animal's midline was set at 45°. Optical imaging was performed transcranially in a rectangular region in the hemisphere contralateral to the stimulated eye, which included the primary visual cortex. (C) Images of blood vessels in the skull and cortical surface (left), and stimulus-evoked intrinsic signals whose strength is indicated by their darkness (right). A detailed analysis was performed using the signals evoked inside the dashed rectangle. The scale bar indicates 1 mm.

We fabricated goggles that were fitted with plano-convex-cylindrical acrylic lenses (height, 8 mm; width, 10 mm; center thickness, 5±0.5 mm; refractive power, 133 diopters; weight, 1.5±0.2 g), as shown in Fig. 5A (left). To mount the goggles on the skull, the mice were first anesthetized with a mixture of ketamine (80 mg/kg) and xylazine (10 mg/kg) i.p. Secondly, an area of the scalp and periosteum of about 5 mm diameter on the frontal bone was resected and the head holder was fixed on the incised part of the area with dental cement (Super Bond; Sun Medical, Shiga, Japan). Thirdly, the frame of the goggles was connected to the head holder with a screw. The position of the goggles was carefully calibrated so that the long axis of the lens was set parallel to the line connecting the medial and lateral canthi, as shown in Fig. 5A (right). Thereby, visual images of the environment were extremely elongated in the vertical direction. Animals were revived after mounting the goggles.

### Surgical pretreatment for in vivo imaging

Mice were anesthetized with a mixture of fentanyl (0.05 mg/kg), midazolam (5.0 mg/kg) and medetomidine (0.5 mg/kg) i.p., and fixed on a stereotaxic frame. Whiskers and scalp fur were removed, and the scalp and periosteum were resected from the lambda to the bregma. The exposed parietal bone was dried and transparentized by coating with a mixture of vaseline and liquid paraffin for intrinsic signal imaging. For two-photon imaging, a cranial chamber for an immersion objective was formed on the skull with dental cement. A small aluminum tube was attached to the chamber, which was clipped with head bars to fix the head of the mouse. To prevent edema of the cortex, dexamethasone (10 mg/kg) was injected s.c. before craniotomy. For dye loading, an area of the skull and dura mater with 2 mm diameter covering the V1 was removed. We placed contact lenses on the eyes with a drop of silicone oil to prevent the eyes from drying. The body temperature was maintained at approximately 37°C and the heart rate and respiration rate were monitored during imaging.

### Intrinsic signal optical imaging

We set the recording area (3.2×4.9 mm) on the occipital cortex so that its center was positioned 1–2 mm in front of the lambda and 2–3 mm laterally from the midline on either the left or right hemisphere (Fig. 5B). The recording area was illuminated with green light (540±5 nm) to capture an image of blood vessel patterns in the skull and cortex (Fig. 5C left), and then with red light (700±10 nm) to record intrinsic signals in response to visual stimuli with a 10-bit CCD camera (CS8310B; Teli, Tokyo, Japan) controlled with an Imager 3001 system (Optical Imaging, Rehovot, Israel).

A CRT or LCD monitor screen for stimulus presentation was placed about 25 cm apart from the animal's left or right eye. The angle between the center of the monitor screen and the animal's midline was set at 45° (Fig. 5B). Visual stimuli of square-wave gratings (spatial frequency, 0.05 cpd; temporal frequency, 2 Hz; contrast ratio, 1/2) drifting back and forth in six orientations (0–150° at intervals of 30°) were produced by a visual stimulus generator (ViSaGe; Cambridge Research Systems, Rochester, UK) and presented to the eye contralateral to the recording hemisphere. The direction of stimulus movement was reversed every 1.5 s. Light stimulation to the ipsilateral eye was prevented using a black contact lens.

The focal plane for imaging was set at a depth of 500 µm below the cortical surface. Images of intrinsic signals were captured at a 30 Hz sampling rate while the animals were exposed to visual stimuli (Fig. 5C right). The dashed outline indicates the region of interest (ROI) whose size is 2.4×3.0 mm, common for all recording sessions on animals examined. Note that the ROI is large enough to cover the mouse V1 [56]. The image data were averaged every 1 s, digitized and stored in a computer. Intrinsic signals were recorded from 1 s before to 5 s after the stimulus onset with an 8 s interval between successive recordings of intrinsic signals. In a single trial, six grating stimuli and a blank stimulus (a uniform gray image) were each presented once in a pseudorandom sequence. Twenty trials were repeated in a single recording session.

### Dye loading and two-photon imaging

A solution of 1 mM Oregon Green 488 BAPTA-1 AM (OGB-1; Invitrogen, Carlsbad, CA) [57] and 0.1 mM Sulforhodamine 101 (SR101; Invitrogen) [58] was filtered through a 0.22-µm-pore filter (Millipore, Billerica, MA). A filamented glass pipette (inner diameter = 0.86 mm; tip diameter <5 µm) was filled with 5 µl of the solution, which was injected into the cortex at a depth of 200–300 µm from the surface under pressure (1–10 psi) for 5–10 min until the dye diffused over a region of diameter 300–400 µm. After the pipette was removed, the exposed cortical surface was filled with 2% agar and sealed with a round coverslip of 4 mm diameter.

A multiphoton laser scanning microscope (FV1000MPE; Olympus, Tokyo, Japan) and a mode-locked Ti:sapphire laser (MaiTai DeepSEE; Newport, Santa Clara, CA) were used for two-photon imaging. The dye was excited by the laser light of 810 nm wavelength with a laser power of less than 80 mW. The emission light was bandpass-filtered to observe fluorescence from OGB-1 (green, 495–540 nm) and SR101 (red, 575–630 nm). An immersion objective lens (×20, 0.5NA) was used. A Fluoview system (Olympus) was used for laser scanning, and images in the recording area (300×300 µm) were recorded with a resolution of 300/512 µm/pixel at a 0.9 Hz sampling rate. Images of 2–4 slices were captured at depths of 100–260 µm from the surface to observe cells presumed to be in layer 2/3.

Six oriented grating stimuli were each presented once for 5 s in a pseudorandom sequence to the eye contralateral to the recording hemisphere using a small LCD monitor in each trial during recording. The interstimulus interval was set at 10 s. Ten trials were performed with a total duration of about 18 min. The Ca^2+^ signals elicited from neurons in response to a stimulus grating of each orientation were recorded in a 15 s peri-stimulation period (2 s before, 5 s during and 8 s after stimulation), and such recording was repeated for the six oriented stimuli over ten trials. During recording, the gap between the cranial chamber and the objective was covered with aluminum foil to shield undesired light from possible external light sources.

### Data analysis of intrinsic signals

All recording data were analyzed using original programs in MATLAB (MathWorks, Natick, MA) according to the following procedure. In each trial, recorded signals in response to the blank stimulus were subtracted from signals in response to grating stimuli to remove the stimulus-unrelated systemic components of signals. Then the signals in the first frame were subtracted from the average signal over the fourth to sixth frames, and the differences were divided by the signals in the first frame to normalize the stimulus-related signals at each pixel. The normalized signals were averaged over all trials for each stimulus orientation to obtain stimulus-related neural responses. After manually removing domains of strong signals elicited from blood vessels, a location of the ROI was automatically determined such that the centroid of stimulus-related responses was placed at the center of the ROI. We confirmed that the ROI of constant size contained all areas exhibiting vigorous responses in animals examined. The mean and standard deviation (SD) of the stimulus-related responses were calculated over the recording area outside the ROI. The mean was subtracted from the stimulus-related responses in the recording area and then normalized by the SD. Thus, the redefined stimulus-related responses produced single-orientation maps, which can be compared among different stimulus conditions and even among different animals.

To obtain the orientation distribution (or histogram), we counted the number of pixels that elicited stimulus-related responses larger than 2SD to each stimulus orientation. Then we normalized the numbers of pixels by the total number of pixels over all stimulus orientations to obtain the relative number of pixels strongly responding to each orientation.

### Data analysis of Ca^2+^ signals

To eliminate low-frequency noise in the Ca^2+^ signals, a high-pass filter (>0.05 Hz) was applied to the recorded signals. First, we discriminated neurons from glial cells based on OGB-1 and SR101 images, and the cell bodies of neurons were identified and manually delineated by circles. Then, the Ca^2+^ signals elicited from identified neurons were averaged over the pixels inside the circles at each frame of a 15 s peri-stimulation period, and the thus-averaged signals in response to each orientation were further averaged over ten trials of recording. The ten trials of the signals were averaged separately for each orientation. The fractional change in fluorescence intensity is defined as 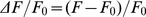, where 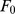 is the mean fluorescence intensity during the prestimulation period and 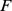 is that for 2 s, during which the strength of the signals were maximal within the stimulation period. The orientation selectivity index (OSI) was estimated using the formula
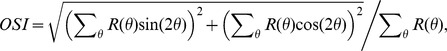
where the response intensity 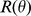 is given by 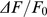 as a function of stimulus orientation 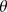. The mean signal strength at each neuron was calculated over the 5 s stimulation period and the six orientations of the stimuli. We defined the baseline signal as the signal elicited 10 s after the onset of stimulation. When response signals greater than the mean signal strength plus 2SD of the baseline signal were sustained for longer than 2 s, we defined such neurons as visually responsive neurons. Visually responsive neurons whose OSIs were greater than 0.1 were defined as oriented neurons. Furthermore, we estimated the density of neurons maximally responding to each orientation to examine the changes in the number of orientation-selective neurons after vertical-orientation exposure.
